# Supplementary material for: The causal relationship between the human gut microbiota and pyogenic arthritis: a Mendelian randomization study
Source: Front Cell Infect Microbiol. 2024 Nov 26;14:1452480. doi: 10.3389/fcimb.2024.1452480 (PMC11629706; doi:10.3389/fcimb.2024.1452480)
Supplement: Supplementary file 1 [file DataSheet1.zip › mendelian test/ebi-a-GCST90027708.csv_leave_one_out.pdf]

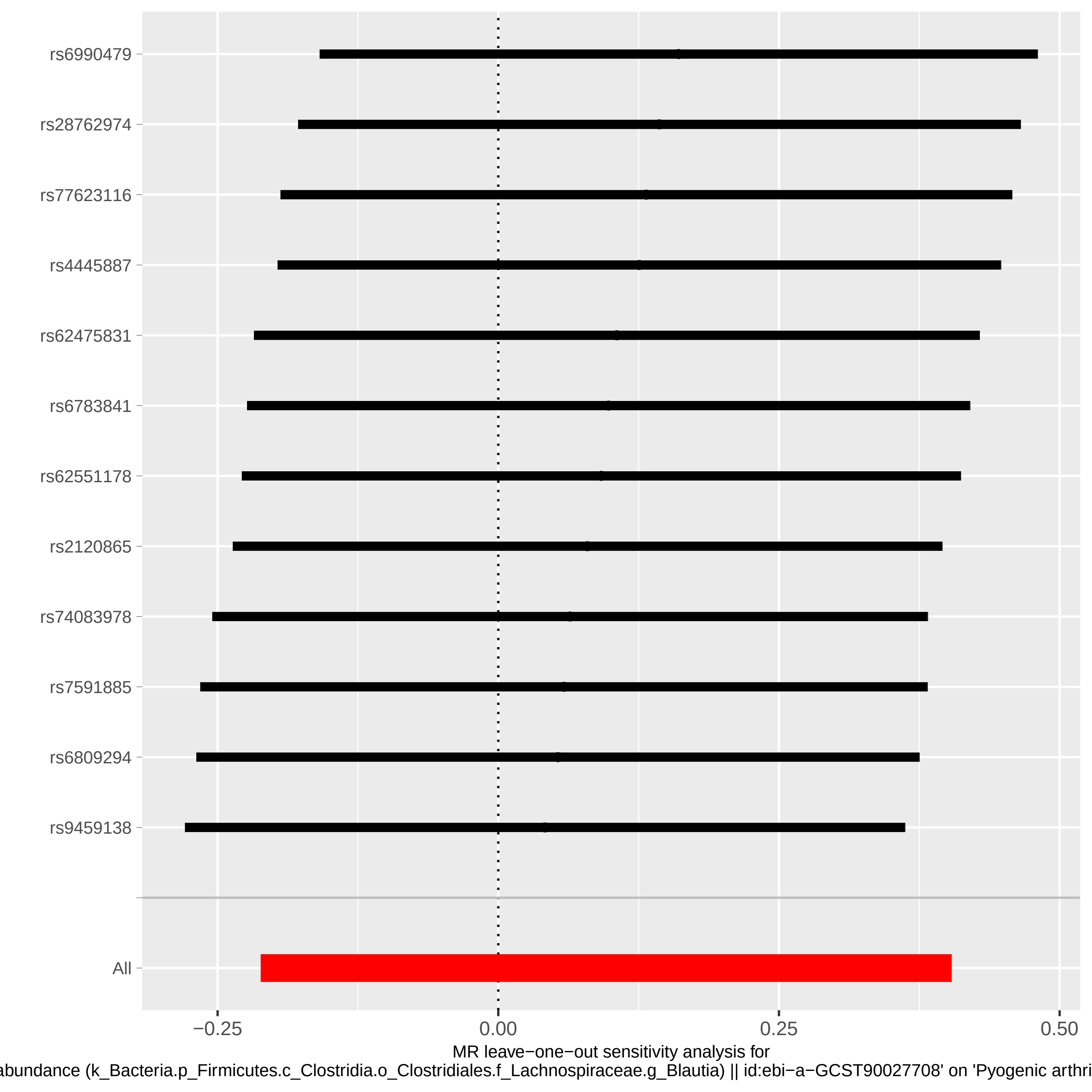

rs6990479
rs28762974
rs77623116
rs4445887
rs62475831
rs6783841
rs62551178
rs2120865
rs74083978
rs7591885
rs6809294
rs9459138
All
−0.25
0.00
0.25
0.50
MR leave−one−out sensitivity analysis for
abundance (k_Bacteria.p_Firmicutes.c_Clostridia.o_Clostridiales.f_Lachnospiraceae.g_Blautia) || id:ebi−a−GCST90027708' on 'Pyogenic arthri
